# Factors Affecting the Perception of Disability: A Developmental Perspective

**DOI:** 10.3389/fpsyg.2021.702166

**Published:** 2021-06-21

**Authors:** Iryna Babik, Elena S. Gardner

**Affiliations:** Department of Psychological Science, Boise State University, Boise, ID, United States

**Keywords:** perception of disability, childhood, development, personality, parental practices, culture

## Abstract

Perception of disability is an important construct affecting not only the well-being of individuals with disabilities, but also the moral compass of the society. Negative attitudes toward disability disempower individuals with disabilities and lead to their social exclusion and isolation. By contrast, a healthy society encourages positive attitudes toward individuals with disabilities and promotes social inclusion. The current review explored disability perception in the light of the in-group vs. out-group dichotomy, since individuals with disabilities may be perceived as a special case of out-group. We implemented a developmental approach to study perception of disability from early age into adolescence while exploring cognitive, affective, and behavioral components of children’s attitudes. Potential factors influencing perception of disability were considered at the level of society, family and school environment, and the individual. Better understanding of factors influencing the development of disability perception would allow the design of effective interventions to improve children’s attitudes toward peers with disabilities, reduce intergroup biases, and promote social inclusion. Based on previous research in social and developmental psychology, education, and anthropology, we proposed an integrative model that provides a conceptual framework for understanding the development of disability perception.

## Introduction

Disability is defined as any impairment of the body or mind that limits a person’s ability to partake in typical activities and social interactions in their environment (Scheer and Groce, 1988). According to the most recent, albeit dated estimates, in the United States, about 16.7% of children have a developmental disability (Boyle et al., 2011), whereas 5.2% of children live with a moderate or severe disability (Brault, 2011; UNICEF, 2013). Since the Individuals with Disabilities Education Act, which mandated inclusive education in 1975, most children with disabilities receive their education in the general education setting, sharing classrooms with typically developing peers (Causton-Theoharis and Theoharis, 2008; U.S. Department of Education, 2012). Interactions between children in such inclusive environments promote acceptance and social inclusion of individuals with disabilities within a classroom and in the society in general (Vignes et al., 2009; de Boer et al., 2013). Social inclusion allows an individual with disabilities to make friends, participate in social activities, and become a contributing and valued member of society (Murray and Greenberg, 2006; Mâsse et al., 2012).

Despite the obvious benefits of inclusive education and social inclusion, children with disabilities are not always accepted by their typically developing peers. Across cultures, children with disabilities encounter negative attitudes, bullying, social exclusion, and isolation (Ochs et al., 2001; Hanvey, 2002; Nowicki and Sandieson, 2002; Cummins and Lau, 2003; Kelly, 2005; Laws and Kelly, 2005; Odom et al., 2006; Guralnick et al., 2007; Shah, 2007; Vreeman and Carroll, 2007; Nugent, 2008; Gannon and McGilloway, 2009; Koster et al., 2010; de Boer et al., 2012a; Lindsay and McPherson, 2012; Snowdon, 2012; Kayama and Haight, 2014). Socially excluded children may have unsatisfying peer relationships, low self-esteem, and lack of achievement motivation, which affect their social and academic aspects of life, mental health, and general well-being (Juvonen and Graham, 2001; Brown and Bigler, 2005; Murray and Greenberg, 2006; Pijl and Frostad, 2010; Lindsay and McPherson, 2012; Mâsse et al., 2012).

Attitudes toward individuals with disabilities vary with the type of disability. For example, children with emotional or behavioral disabilities and those with multiple disabilities are perceived more negatively by their typically developing peers than children with a specific physical disability (McCoy and Banks, 2012). Moreover, children with intellectual or physical/intellectual disability are perceived more negatively than children with a physical disability (Nowicki, 2006; de Laat et al., 2013), with level of social inclusion being positively related to the mental age of the child with disability (Carvalho et al., 2014). In the school context, with its high expectations to learn and negative future consequences of failing to do so, intellectual disability may have greater salience to typically developing children than physical disability.

Children with positive attitudes toward peers having disabilities may be more willing to interact with them compared to children with negative attitudes (Diamond, 1993; Okagaki et al., 1998; Roberts, 1999; Roberts and Smith, 1999; Favazza et al., 2000; Gaad, 2004). As a result, more exposure to individuals with disabilities may lead to better understanding of disability and higher levels of acceptance (Hong et al., 2014). Thus, attitudes drive behavior, which, in turn, affects the individual’s knowledge, beliefs, and attitudes. Interventions improving children’s knowledge about disabilities and providing exposure to those with disabilities is the most successful technique of changing children’s attitudes toward peers with disabilities (Diamond and Carpenter, 2000; Nikolaraizi et al., 2005; Nowicki, 2006; Rillotta and Nettelbeck, 2007; Siperstein et al., 2007; Feddes et al., 2009; Kalyva and Agaliotis, 2009; Gasser et al., 2014; Armstrong et al., 2016). Developmental psychologists suggest that early childhood is the best time to intervene against the formation of negative attitudes toward disability, before these attitudes and behavior patterns become fully established and difficult-to-change (Killen et al., 2011; Lee et al., 2017).

The main goal of the current review was to explore factors influencing the formation of attitudes toward disability during childhood, and identify developmental trends that produce negative attitudes toward disability in typically developing children. Knowledge about these trends is important for designing timely, age-appropriate, and effective interventions to reduce the behaviors of stigmatization and social exclusion (Abrams and Killen, 2014; Kayama, 2017). In addition to studying the development of attitudes toward individuals with disabilities, the current review examined the cognitive, affective, and behavioral^1^ aspects of attitudes (Cook and Selltiz, 1964; Triandis, 1971; Olson and Zanna, 1993; Findler et al., 2007). Also, this review evaluated personality factors, family influences, as well as cultural norms and traditions in order to better understand the full context of these attitudes (Bronfenbrenner, 1992; Bronfenbrenner et al., 1994).

## Conceptual Models of Disability

Two competing conceptual models of disability have been used to define the origins of the abnormal physiological and psychological functioning (LoBianco and Sheppard-Jones, 2008). The ***medical model*** considers disability a feature of the person, directly caused by diseases, disorders, traumas, or other health conditions, which would require medical treatment or intervention with the primary goal to “correct” the problem within the individual (Johnston, 1996; Marks, 2000; Mitra, 2006; Forhan, 2009; Nind et al., 2010; Brandon and Pritchard, 2011; Palmer and Harley, 2012; Bingham et al., 2013).

By contrast, the ***social model*** does not consider the disability an attribute of the individual, but rather a socially created problem (Hutchison, 1995; Mitra, 2006; Purdue, 2009; Barney, 2012). In this case, the problem that needs to be corrected lies not within the individual, but within the unaccommodating social environment (Brandon and Pritchard, 2011; Roush and Sharby, 2011; Barney, 2012; Palmer and Harley, 2012; Bingham et al., 2013). According to the social model, disability could be imposed by society on individuals with impairments through isolation and exclusion from everyday activities (Brandon and Pritchard, 2011; Bingham et al., 2013). Such isolation and exclusion may stem from society’s unfavorable perceptions of people with disabilities and unwillingness to remove environmental barriers impeding full participation (LoBianco and Sheppard-Jones, 2008; Forhan, 2009; Palmer and Harley, 2012).

However, neither medical nor social model acknowledge the complex nature of disability. Therefore, a comprehensive integration of the two approaches produced the ***biopsychosocial model***, which considers disability in the context of an interaction between biological, psychological, and societal factors, each limiting the individual’s functioning to some extent (Engel, 1980; Borrell-Carrió et al., 2004; Thomas, 2004; Shakespeare, 2006; Le Boutillier and Croucher, 2010). In the light of this model, the World Health Organization defined disability as “the outcome or result of a complex relationship between an individual’s health condition and personal factors, and of the external factors that represent the circumstances in which the individual lives” (Peterson, 2005, p. 106). Importantly, the extent to which impairment becomes a disability depends not only on the severity of the impairment, but also on the individual’s ability to participate in social life (Hall and Hill, 1996; Peterson, 2005).

The biopsychosocial model can be viewed as an implementation of the ***ecological systems theory*** (Bronfenbrenner, 1992; Bronfenbrenner et al., 1994) in the context of disability. Indeed, this theory examines ways the synergistic interaction between characteristics of the individual and features of the environment produces the individual’s behavior and development. Functioning of the individual with disability in the society, as well as the perception of this individual by other members of the society may depend on an array of factors, such as the type and severity of disability, personality traits of the individual, available physical environment adaptations, financial resources, social inclusion practices, parental attitudes and practices, availability of inclusive education, teachers’ attitudes and ability to scaffold positive interactions between students, cultural beliefs and traditions, as well as the historical context.

## In-Group vs. Out-Group Perceptions and Attitudes

People tend to view others as belonging to either a familiar in-group or an unfamiliar out-group (Allport, 1954; Hatemi et al., 2013). The out-group may consist of any individuals not belonging to the in-group; thus, racial minorities, sexual minorities, immigrants, and people with disabilities often are perceived as out-groups. The emotionally loaded process of groups’ juxtaposition may result in a biased, more favorable perception of the in-group in comparison to out-groups (Tajfel, 1982; Brewer and Kramer, 1985; Tajfel and Turner, 1986; Devine, 1995; Gramzow and Gaertner, 2005).

Developing social identity shapes the individual’s self-beliefs and determines one’s place in relation to others (Tajfel and Turner, 1979). To protect and promote the self through the in-group, the individual may be motivated to over-value the in-group and derogate an out-group (Tajfel and Turner, 1979; Brewer, 1999; Aboud, 2003; Dovidio et al., 2010). Thinking in terms of “us” vs. “them” leads people to perceive an out-group as a potential threat (Stephan and Stephan, 1985). For example, members of an out-group may have different values and beliefs, may potentially disapprove of and reject the in-group, or may undermine the power of the in-group in the political, economic, or cultural domains (Esses et al., 1993; Stephan and Stephan, 1993; Quillian, 1995). This perceived threat may provoke negative expectations about, and reactions to, out-groups, including stigmatization and discrimination, as well as a desire to protect the in-group (Stephan and Stephan, 1993; Ybarra and Stephan, 1994). The resulting intergroup biases lead to social exclusion of out-groups by members of the in-group. Note that intergroup biases may be activated by explicit mentioning of intergroup norms or potential out-group threat (Blanchard et al., 1994; Monteith et al., 1996; Fahmy et al., 2006).

Individuals differ in their dispositional reaction toward potential threats posed by out-groups. Some exhibit a negativity bias while avoiding interactions, whereas others tend to respond in a more approach-oriented manner (Hibbing et al., 2014). Previous research found a strong association between individuals’ dispositional reaction to potential threats and their political views. Thus, individuals with conservative views tend to avoid uncertainty in order to reduce possible negative outcomes, while those with liberal views tend to approach the threatening stimulus in hopes to engender positive change (Jost et al., 2003; Hibbing et al., 2014; Hatemi and McDermott, 2020). For example, when being exposed to pictures with ambiguous emotional expressions, self-reported conservatives perceive them to be angry and potentially threatening, whereas liberals perceive them as being confused and non-threatening (Vigil, 2010). As a result, the negativity bias may lead people to discriminate against (socially exclude) members of an out-group as a source of uncertainty and potential threat.

Note that the negativity bias was observed not only on a psychological, but also on a physiological level. Thus, people who tend to protect the in-group against out-groups (those promoting military defense and anti-immigration policies), when being presented with threatening stimuli or images associated with out-groups, show greater attention to the threat (Nail et al., 2009), as well as greater physiological arousal and sympathetic nervous system activity, measured via skin conductance (Antony et al., 2005; Oxley et al., 2008; Dodd et al., 2012; Hatemi et al., 2013; Renshon et al., 2015; Garrett, 2019). Therefore, uncertainty and perceived threat may elevate levels of fear and anxiety in some individuals, making them less willing to embrace novel social situations or interact with new people, and be more intolerant toward members of an out-group (Jost et al., 2003; Hatemi et al., 2013).

For this review, it is also important to distinguish between peer rejection and out-group exclusion. Peer rejection is often attributed to the individual characteristics and behavior of the rejected person, making the victim the source of the exclusion. By contrast, out-group exclusion arises from internal insecurities of the excluding individual, social attitudes, group norms, stereotypic expectations, and intergroup biases (Killen et al., 2013). Unfortunately, it is often hard to distinguish the cause from the consequence in this complicated, dynamic process. For example, a child with disability may be socially excluded by peers as a member of an out-group; this experience may result in this child becoming socially withdrawn, timid, and shy; such attributes, in turn, would seemingly justify the resulting peer rejection based on personality characteristics.

## Development of Disability Perception During Childhood

During early development, as children integrate into society and become members of social groups, they develop not only social identity and bonds with family and peers, but also social preferences, prejudices, and intergroup biases. Children under the age of ***3 years*** show social preferences for individuals based on age, gender, language, and other salient characteristics, such as, for example, a T-shirt color (LaFreniere et al., 1984; Aboud, 1988; Martin, 1989; Kinzler et al., 2007; Shutts et al., 2010; Dunham et al., 2011). From the age of 3 years onward, children tend to display a positivity bias – expecting positive personality characteristics in novel individuals and focusing on positive information about self and others (Mezulis et al., 2004; Boseovski and Lee, 2006; Boseovski et al., 2009; Boseovski, 2012; Landrum et al., 2013; Lapan et al., 2016). In general, 3-year olds make preferential judgments about other individuals based on similarity, whether in appearance or food preferences (Fawcett and Markson, 2010). In these social judgments, 3-year olds seem to focus on individual characteristics rather than group affiliations. Thus, being randomly assigned to an arbitrary, minimal social group^2^, 3-year olds remembered their affiliation with the group, but displayed no sociocentric reasoning, or in-group vs. out-group biases (Dunham and Emory, 2014).

Around the age of ***4 years***, children start manifesting in-group positivity bias, seemingly extrapolating self-related positivity toward groups they affiliate with (Gramzow and Gaertner, 2005). Thus, children attribute more positive characteristics toward in-group members compared to out-group ones (Bigler and Liben, 1993; Aboud, 2003; Kinzler et al., 2009; Hilliard and Liben, 2010; Cvencek et al., 2011; Dunham et al., 2011; Renno and Shutts, 2015; Over et al., 2018); they show preference toward their own gender and race (Hilliard and Liben, 2010; Cvencek et al., 2011; Renno and Shutts, 2015), as well as toward their own, non-accented language (Kinzler et al., 2009). It is still easy for children at this age to accept peers with disabilities, likely because of the low-level of complexity in their activities (Hestenes and Carroll, 2000). In later ages, however, there may be a more distinct disconnect in physical, cognitive, and socioemotional abilities between typically developing children and their peers with disabilities (Gasser et al., 2014).

Experimental studies reported that ***5–6-year*** olds are capable of negative attitudes toward out-group members (McLoughlin and Over, 2017; McLoughlin et al., 2017). While tested in the minimal group paradigm, 5–6-year olds not only internalized their membership in a minimal group, but also developed a predisposition to prefer the in-group and evaluate it more favorably than an out-group (Aboud, 2003; Baron and Banaji, 2006; Rutland et al., 2007; Dunham et al., 2011; Buttelmann and Böhm, 2014; Dunham and Emory, 2014; Baron and Dunham, 2015). Moreover, 6-year olds showed positive affect after mere exposure to in-group member photographs, and assumed that in-group members would be less likely to engage in negative actions compared to out-group peers (Nesdale and Brown, 2004; Dunham and Emory, 2014; Baron and Dunham, 2015). Such in-group favoritism becomes more salient in indirect measures rather than self-reports, suggesting operation of an automatic, implicit evaluative system (Dunham and Emory, 2014).

Intergroup biases become even more pronounced in ***6–7-year*-**old children. Even when 6–7-year olds display a positivity bias while accepting positive testimonies about in-group and out-group members, this bias is disproportionately higher in the case of the in-groups (Aldan and Soley, 2019). Also, while choosing to accept or reject someone’s testimony about novel individuals, 6–7-year olds tend to trust an in-group informant more than an out-group one, especially when evaluating novel out-group individuals (Kinzler et al., 2011; Aldan and Soley, 2019). This over-reliance on in-group informants during the evaluation process of novel individuals may further exacerbate the emerging intergroup biases. Thus, the developmental evidence suggests that children from 5 to 9 years of age tend to learn new information about novel members through the prism of the established intergroup biases (Averhart and Bigler, 1997; Nesdale and Brown, 2004; Dunham et al., 2011; Baron and Dunham, 2015).

In summary, between the ages of 3 and 6 years, children’s social awareness shifts from being individuals to being members of a social group (Dunham and Emory, 2014). Increasing familiarity with individual characteristics of the immediate family and the surrounding social circle makes children aware of multiple ways people are grouped in the society. Children’s experiences of being affiliated with, or rejected from, particular groups shape their sociocentric awareness and social cognition about in-groups vs. out-groups (Aboud, 1988; Dunham and Emory, 2014; Nesdale et al., 2014). This social cognition uses the heuristics of “us” vs. “them” to automate social judgments; such automation of the evaluative system, though, comes at the cost of intergroup biases (Bigler and Liben, 2007; Dunham and Emory, 2014). Thus, while becoming integrated into society, children first manifest social awareness and form group identity (3–5 years of age), then show in-group preference and in-group positivity (4–6 years of age), and finally display out-group prejudice and out-group derogation (by about 7 years of age) (Brewer, 1999; Aboud, 2003; Nesdale, 2004, 2008).

Between ***6 and 9 years*** of age, children experience a dramatic shift in their self-identity, which instead of being focused on group membership, becomes focused on group norms (Abrams and Rutland, 2008). Thus, older children practice social exclusion based on the norms of the in-group. As a result, stronger self-identity and affiliation with the in-group, emphasis on the group membership, explicitly articulated negative messages about out-groups, expression of exclusion norms, and perceived threat from an out-group are associated with an increase in intergroup biases and stronger negativity toward out-group members in 6–11-year olds (Bigler et al., 1997; Nesdale et al., 2005a,c; Nesdale and Dalton, 2011; Nesdale and Lawson, 2011; Durkin et al., 2012). Importantly, developing in socially homogeneous environments may speed up the emergence of negative biases toward out-groups, resulting in early onset between ages 3 and 5 years (Rutland et al., 2005a). On the other hand, knowledge about out-groups and exposure to out-group members may allow children to include those in their own self-concept, resulting in inclusion and positive attitudes (Wright et al., 1997).

During childhood, the development of intergroup biases seems to have an inverted U-shape: generally positive attitudes of 3-year-old children become increasingly negative by the age of 7–8 years, with negativity decreasing thereafter (for review, see Raabe and Beelmann, 2011). Depending on the context, this general timeline may shift either way. For example, some researchers reported a decrease in negative attitudes toward children with intellectual disabilities from the age of 4–10 years (Nowicki, 2006). It was suggested that younger children may over-generalize the situation and take into account only most salient characteristics of an evaluated individual, whereas older children are capable of analyzing a situation from multiple perspectives and considering a complex array of factors (Magiati et al., 2002). Moreover, with age, children learn to rely more on their experience rather than external instruction. For example, in a minimal group paradigm, when a negative, overt message contradicted their own positive personal experience with an out-group, 6–7-year olds relied on the external instruction for their out-group evaluation, whereas 10–11-year olds trusted their own experience (Kang and Inzlicht, 2012).

Importantly, children’s social development in the form of social attributions and in-group biases depends on their knowledge about different disabilities, understanding of disability, as well as general cognitive development (Magiati et al., 2002; Diamond and Huang, 2005; Diamond et al., 2008; Diamond and Hong, 2010; Gasser et al., 2014). According to Piaget (1970), children at the age of 2–7 years are at the ***preoperational stage*** of cognitive development; their thinking is perception-based and symbolic; they typically attend to the most salient features, while ignoring less obvious attributes or the situational context. Thus, while evaluating peers with disabilities, typically developing 5-year olds tend to consider only the highly noticeable features of an individual, such as adaptive equipment, while disregarding less noticeable features related to the individual’s dyslexia, hyperactivity, intellectual disability, or autism (Conant and Budoff, 1983; Favazza and Odom, 1997; Diamond and Kensinger, 2002; Magiati et al., 2002). Therefore, not surprisingly, young children have better understanding of physical disabilities compared to intellectual ones (Magiati et al., 2002; Laws and Kelly, 2005; Diamond et al., 2008; Diamond and Hong, 2010). Young children’s heightened attention to saliency may have another negative effect: the use of salient identifying labels for an out-group can trigger intergroup bias in 3–5-year-old children (Patterson and Bigler, 2006; Bigler and Liben, 2007; Hilliard and Liben, 2010).

At the age of 7–11 years, children are at the ***concrete operational stage*** of cognitive development; they begin thinking logically, decrease their overgeneralizing tendency (Gasser et al., 2014), and are more capable of analyzing a situational context from multiple perspectives. Better understanding of disability makes typically developing children more likely to engage in play activities with peers having disabilities (Diamond and Huang, 2005). Better knowledge about and understanding of disability also allows typically developing children to overcome a tendency to generalize deficits across different domains (e.g., assume that a child in a wheelchair would also be less cognitively competent) and, instead, select activities that do not involve the affected domains and allow a child with disability to fully participate (Diamond et al., 1997; Diamond and Hong, 2010; Gasser et al., 2014). Better understanding of disability, as better understanding of any out-group, reduces fears about this group and facilitates positive attitudes (Katz and Chamiel, 1989; Okagaki et al., 1998).

Another important factor facilitating more positive attitudes toward individuals with disabilities is children’s ability to engage in moral reasoning when justifying social inclusion (Fisher et al., 1998; Turiel, 1998; McDougall et al., 2004; Smetana, 2006; Gasser et al., 2014; Beaulieu-Bergeron and Morin, 2016; Shalev et al., 2016). Moral reasoning incorporates concepts of fairness, justice, equality, and human rights into social evaluations (Killen and Rutland, 2011). Being reminded about fairness and equality, even 3–5-year-old typically developing children show improved inclusion of children with disabilities (Diamond and Tu, 2009; Diamond and Hong, 2010). Explicit education about prejudice, intergroup biases, and social justice reduces intergroup biases in 6–13-year-old children (e.g., Aboud and Doyle, 1996; Aboud and Fenwick, 1999; Hughes et al., 2007; Brinkman et al., 2011). Note that ***explicit intergroup biases*** decrease with age due to social desirability concerns, as children (by about the age of 8 years) become aware of social norms explicitly condemning prejudiced social judgments and become motivated to conform to those norms (Rutland et al., 2005b; FitzRoy and Rutland, 2010). ***Implicit intergroup bias***, on the other hand, seems to be unaffected by social desirability pressures, likely due to the lack of public accountability (Rutland et al., 2005b; Skinner and Meltzoff, 2019).

## Personality Factors Affecting Perception of Disability

Roots of intergroup biases and social exclusion can be traced to early developing personality traits, as well as the features of the individual’s social-emotional and social-cognitive development.

### Temperament

Previous research points toward continuity in the development of personality traits from early childhood into adulthood. Thus, personality traits exhibited by children during preschool years are positively correlated with those manifested during young adulthood. Importantly, these personality traits to a large extent determine the person’s beliefs, attitudes, and behaviors.

For example, self-confident, autonomous, resilient, expressive, impulsive, and social 3–4-year-old children became open-minded, approach-oriented, and novelty-seeking adults (Jost et al., 2003; Carney et al., 2008; Janoff-Bulman et al., 2008; McAdams et al., 2008; Mondak and Halperin, 2008; Gerber et al., 2010; Kantner and Lindsay, 2014). As adults (at the age of 23 years), they expressed liberal views, while welcoming novelty, embracing change, denouncing social inequality, showing greater openness toward out-groups and less propensity toward worldview defense (Mikulincer and Florian, 2000; Mikulincer and Shaver, 2001; Jost et al., 2003; Block and Block, 2006; Oxley et al., 2008; Mondak, 2010; Fraley et al., 2012).

By contrast, fearful, indecisive, withdrawn, inhibited, rigid, and easily victimized children became timorous, uncomfortable with uncertainty, loving structure and order, rigid adults (Van Hiel and Mervielde, 2004; Block and Block, 2006; Jost et al., 2007). As adults, such individuals supported conservative views, promoting traditional values, established modes of behavior, strict rules, domestic surveillance, resistance to change, restricted immigration, and acceptance of inequality (Jost et al., 2003; Block and Block, 2006; McAdams et al., 2008; Janoff-Bulman, 2009; Wegemer and Vandell, 2020). Thus, early temperament may facilitate the development of personality traits that would promote or impede the formation of intergroup biases and negative attitudes toward out-group members in general and individuals with disabilities in particular.

### Empathy and Sympathy

Empathy and sympathy are critical for the development of prosocial behavior, social competence, and moral reasoning (Diamond, 2001; Eisenberg et al., 2006; Mestre et al., 2019; Portt et al., 2020). Empathy is the ability to feel and understand another person’s emotional state or condition through emotional matching and affect sharing (Eisenberg et al., 2006; Cuff et al., 2016). Sympathy is an emotional response to another person’s troublesome situation, typically expressed as feelings of pity, sorrow, or concern for the other (Eisenberg et al., 2006).

Caregivers are children’s first teachers of empathy – they often mirror their infants’ positive and negative emotions, such as happiness, surprise, anger, and sadness (Tronick, 1989; Gergely and Watson, 1996; Ray and Heyes, 2011; Heyes, 2018). In response, infants try to imitate caregivers’ facial expressions associated with certain emotions, and, by doing this, gradually internalize the emotional experiences of others (Atkinson, 2007; McDonald and Messinger, 2011). For example, mirroring its mother’s smile may bring an infant a feeling of happiness; thus, mimicking facial expressions gradually transforms into sharing the other’s emotional state and, eventually, into emotional empathy.

Previous research found a significant, albeit gender-stereotyped, relation between children’s and parents’ empathy and sympathy: the child’s empathy and sympathy are related to the corresponding attributes in the same-sex parent (Barnett et al., 1980; Eisenberg et al., 1991; Eisenberg and McNally, 1993). Development of empathy in children seems to be advanced by parents’ ability to be empathetic of their children’s emotions. Furthermore, regular observations of parent’s empathic reactions toward oneself make children more likely to model such empathic behaviors in their own interactions with others, thus, reinforcing their empathic skills. The quality of parent–child relationships (e.g., parental warmth and responsiveness, secure attachment^3^, parent–child synchrony, shared positive affect, parental use of reasoning) is positively related to children’s and adolescents’ empathy and sympathy levels, as well as their tendency toward prosocial behaviors (Kestenbaum et al., 1989; Janssens and Gerris, 1992; Staub, 1992; Krevans and Gibbs, 1996; Kochanska, 2002; Van der Mark et al., 2002; Zhou et al., 2002; Kiang et al., 2004; Davidov and Grusec, 2006; Spinrad and Stifter, 2006; Feldman, 2007; Moreno et al., 2008).

Children’s greater empathy results in a better ability to understand others’ feelings and a higher likelihood of responding in a more appropriate, sensitive manner and genuinely trying to help; the latter behavioral patterns result in more positive social interactions. Indeed, both empathy and sympathy are positively related to the quality of interpersonal relationships, prosocial behaviors (e.g., caring for others, working to relieve suffering, treating others with kindness), and moral reasoning in children and early adolescents (Eisenberg and Miller, 1987; Eisenberg and Fabes, 1990; Zahn-Waxler et al., 1995; Hoffman, 2000; Eisenberg et al., 2006; Knafo et al., 2008; Stocks et al., 2009; Mestre et al., 2019; Portt et al., 2020).

Empathy and sympathy are also associated with social competence measures, such as peer sociocentric status, perspective taking, cooperation, conflict resolution skills, as well as socially appropriate behaviors (Adams, 1983; Eisenberg and Miller, 1987; Eisenberg and Fabes, 1995, 1998; Eisenberg et al., 1996; Zhou et al., 2002; Sallquist et al., 2009; Carlo et al., 2010). Empathy and sympathy direct a person’s attention toward others’ feelings, situation, and needs; this other-oriented approach inhibits aggressive responses, motivates non-egoistic prosocial behavior, and facilitates the development of moral reasoning (Eisenberg, 1986; Miller and Eisenberg, 1988; Batson, 1991; Hoffman, 2000; Eisenberg et al., 2001).

Empathy-associated decrease in aggressive behavior may inhibit bullying tendencies (Kaukiainen et al., 1999; Albiero and Lo Coco, 2001). Indeed, starting at the age of 6 years old, high levels of empathy and sympathy are associated with low levels of children’s aggression and bullying behavior, as well as a higher likelihood of defending a victim (Cohen and Strayer, 1996; Warden and Mackinnon, 2003; Strayer and Roberts, 2004; Mayberry and Espelage, 2007; Gini et al., 2008; Stavrinides et al., 2010; Barchia and Bussey, 2011; Jolliffe and Farrington, 2011). By contrast, a lack of empathy and sympathy may negatively affect children’s socioemotional development and result in bullying behavior. This trend continues into adolescence: low empathy is related to bullying behavior in 13–16-year olds (Endresen and Olweus, 2002; Jolliffe and Farrington, 2006).

Although previous research reported that both affective (feeling others’ emotions) and cognitive (understanding others’ emotions) components of empathy are negatively associated with bullying (Mitsopoulou and Giovazolias, 2015), the cognitive aspect has a much weaker effect (Bryant, 1982; Cohen and Strayer, 1996; LeSure-Lester, 2000; Jolliffe and Farrington, 2006). Importantly, empathy-engendered prosocial behavior in peer-to-peer interactions may increase children’s positive attitudes toward members of out-groups and peers with disabilities. Previous research identified a bidirectional relation between emotional sensitivity and attitude toward individuals with disabilities: positive interactions with peers having disabilities make children more conscious of others’ emotional states and, therefore, more accepting of peers with disabilities; by contrast, limited exposure to peers with disabilities is associated with lower levels of both emotional sensitivity and disability acceptance (Diamond, 2001; Diamond et al., 2008; Yu et al., 2015).

### Theory of Mind

Theory of mind (ToM) is defined as the ability to understand that others’ perspective, knowledge, beliefs, thoughts, and intentions may differ from one’s own (Wellman, 1990; Frith and Frith, 2005; Eisenberg et al., 2006). ToM is sometimes referred to as ***cognitive empathy*** (McDonald and Messinger, 2011). Using false belief tests^4^, developmental researchers found that by about 4 years of age children are capable of seeing a situation from the perspective of others, making inferences about the beliefs and intentions of others, and interpreting others’ behavior in the light of those beliefs (Wellman, 1991; Wellman and Bartsch, 1994; Wellman et al., 2001). Further improvement of ToM skills continues during the preschool years (Wellman et al., 2001); older age is associated with more advanced ToM skills (Walker, 2005; Lapan and Boseovski, 2016); girls reportedly develop ToM sooner than boys (Walker et al., 2002; Walker, 2005).

Children’s understanding of other people’s circumstances and needs allows better perspective taking, more effective helping strategies, cooperative play behavior, better conflict-management skills, positive interactions with peers, more prosocial behaviors, and higher social competence (Dunn et al., 1991; Dunn and Cutting, 1999; Watson et al., 1999; Jenkins and Astington, 2000; McDonald and Messinger, 2011; Caputi et al., 2012). By contrast, lack of ToM is associated with difficulties interpreting social information, less positive social interactions, and underdeveloped social skills (Mundy and Crowson, 1997; Lapan and Boseovski, 2016). Note that there is a bidirectional relation between the ToM skills and successful social interactions: while better ToM skills promote more positive social interactions, the latter, in turn, improve the child’s ToM (Watson et al., 1999).

Previous research found a significant relation of the ToM level to children’s perceptions and trait attributions of typically stigmatized individuals (Lapan and Boseovski, 2016). Sensitivity to others’ ***internal*** states (beliefs, emotions, and intentions) makes their ***external*** characteristics less salient (Lapan and Boseovski, 2016). Therefore, well-developed ToM skills enable children to appreciate individual differences and correctly evaluate others’ beliefs and abilities within the situational context (Miller, 2002; Diamond and Hong, 2010). In this case, children with disabilities would be viewed in the light of their internal dispositions rather than, for example, visible orthosis or a wheelchair. Indeed, advanced ToM skills are associated with less hostile, more positive or neutral, and more sophisticated attributions of typically stereotyped characters; as well as more positive behavioral predictions about them (Weiner et al., 1982; Thompson, 1989; Erdley and Dweck, 1993; Choe et al., 2013; Lapan and Boseovski, 2016). As a result, children with well-developed ToM skills are more likely to include a child with physical disability into play activities after appropriate evaluation of the task demands and the child’s previous experience (Diamond and Hong, 2010).

Importantly, well-developed ToM skills result in the ability to regulate explicitly biased attributions and internalize bias reduction: whereas public settings with high public accountability makes all children exhibit more positive trait attributions, only children with higher level of ToM skills show positive attributions in a private setting with low public accountability (Gee and Heyman, 2007; FitzRoy and Rutland, 2010; Aboud, 2013; Nesdale, 2013; Rutland, 2013; Beelmann and Heinemann, 2014). Thus, children with well-developed ToM skills are more likely to contemplate the legitimacy of their negative attributions and possible consequences of making potentially incorrect or offensive attributions about individuals with disabilities (Lapan and Boseovski, 2016).

### Self-Esteem

Individuals’ self-esteem is another important factor influencing attitudes toward out-groups and people with disabilities. Self-esteem defines the extent to which an individual approves of, likes, and values oneself (e.g., Blascovich et al., 1991). Evaluation of others and behaviors toward them start with the evaluation of self, and, thus, other-evaluation may be explored through the prism of self-esteem. The ability to satisfy the fundamental need to belong through positive social interactions with others improves the individual’s self-esteem (Baumeister and Leary, 1995; Leary and Baumeister, 2000). On the other hand, adequate self-esteem is associated with better mental health and more positive interpersonal dynamics (Greenberg et al., 1992; Denissen et al., 2008). Thus, there is a bidirectional link between self-esteem and attitudes/behaviors toward others.

Quality of the parent-child relationships is positively related to children’s and adolescents’ self-esteem and social competence (Simons and Robertson, 1989; Riggio et al., 1990; Allen et al., 1994; Arbona and Power, 2003; Kim and Cicchetti, 2004). For example, children of supportive parents, who encourage independence, are more likely to have a high self-esteem and better social skills (Riggio et al., 1990; McCormick and Kennedy, 1994). Also, being securely attached may serve as a protective factor for self-esteem: priming a person with a secure base (exposure to the name of a supportive other) leads to a more positive self-evaluation (Baldwin, 1994). Attachment to the in-group has a similar protective effect: in-group membership allows individuals to maintain high self-esteem through intergroup comparisons that favor the in-group and often devalue members of out-groups (Tajfel and Turner, 1986; Crocker and Luhtanen, 1990; Hogg and Abrams, 1990; Mikulincer and Shaver, 2001).

Previous research experimentally manipulated the perceived threat to a person’s self-esteem in order to evaluate how that affected their behavior toward others. Exposure to false negative feedback, which signals failure and threatens the person’s self-esteem, increases authoritarian^5^ responses and negative reactions toward out-groups; by contrast, a false positive feedback results in lower authoritarian tendencies and more positive attitudes toward others (Sales and Friend, 1973; Fein and Spencer, 1997). High self-esteem is associated with more advanced social skills. For example, high self-esteem reportedly protects children and adolescents from involvement in bullying, both as victims or bullies (O’Moore and Hillery, 1991; Byrne, 1994; Rigby and Cox, 1996; O’Moore and Kirkham, 2001; but also see Olweus, 1993; Slee and Rigby, 1993; Kaukiainen et al., 2002). Importantly, children with better self-beliefs concerning their social competence have more positive attitudes toward peers with disabilities (Hellmich and Loeper, 2019).

### Gender Differences

Gender has been considered one of the factors potentially influencing perception of disability, although previous research on this topic produced mixed findings. Some studies showed no difference between self-identified boys and girls in their attitudes toward peers with disabilities (Tamm and Prellwitz, 2001; Nikolaraizi et al., 2005; Hong et al., 2014). However, most studies reported that 4–14-year-old girls manifest more positive attitudes and higher levels of acceptance toward children with disabilities compared to boys (Sigelman et al., 1986; Nikolaraizi and De Reybekiel, 2001; Laws and Kelly, 2005; Nowicki, 2006; Siperstein et al., 2007; Diamond et al., 2008; Vignes et al., 2009; Gökbulut et al., 2017; Ersan et al., 2020).

In terms of the disability type, 9–12-year-old girls, compared to boys, showed more positive attitudes toward children with hearing or visual impairments, as well as those with physical impairments; whereas no gender differences were found in children’s attitudes toward peers with behavioral difficulties (Nikolaraizi and De Reybekiel, 2001; Laws and Kelly, 2005). In terms of the attitude components, Nowicki (2006) found that 4–10-year old girls were more accepting toward children with disabilities than boys, but only on the cognitive, and not on the emotional or behavioral levels. By contrast, Armstrong et al. (2016) reported that 7–16-year-old girls demonstrated more positive affective and behavioral components of attitudes than boys.

Interestingly, Nowicki (2006) reported more positive attitudes of girls compared to boys toward any targets: peers without disability, as well as peers with physical, intellectual, or physical and intellectual disability. This indiscriminate positivity may reflect a gender-specific response bias rather than actual gender differences in attitudes toward children with disabilities. Girls’ positivity could be attributed to their greater emotional sensitivity, compassion, empathy, or tendency toward prosocial behavior (Walker, 2005; Han et al., 2006; Landazabal, 2009; Gökbulut et al., 2017), all of which likely being the result of traditional differences in social norms and expectations, as well as socialization practices between boys and girls (Walker, 2005).

## Parental Factors Affecting Perception of Disability

Family plays a significant role in shaping children’s beliefs and attitudes toward others: parenting styles and children’s attachment styles may determine the child’s future attitudes toward individuals with disabilities. Importantly, there is an intricate interplay between parental factors and children’s personality factors.

### Parental Influences

Being the primary agents integrating children into society, parents may significantly influence their children’s attitudes toward out-groups in general and individuals with disabilities in particular (Hellmich and Loeper, 2019). However, previous research showed inconsistent findings relating parents’ and children’s beliefs about people with disabilities: some found positive relation (Katz and Chamiel, 1989; Peck et al., 1992; Okagaki et al., 1998; Innes and Diamond, 1999; Vignes et al., 2009; de Boer et al., 2011, 2012b; Hellmich and Loeper, 2019), while others found no relation (Aboud and Amato, 2001; Perkins and Mebert, 2005; Vittrup and Holden, 2011; Pahlke et al., 2012; Hong et al., 2014; Jugert et al., 2016).

Importantly, parents may communicate their beliefs and attitudes to children ***explicitly*** – through discussions or explicit teaching, or ***implicitly*** – by modeling their values in daily interactions with other people or by providing their children opportunities to interact with out-group peers (Dunn, 1993; Castelli et al., 2007; Hellmich and Loeper, 2019). While this differentiation is important, it still does not lead to consensus. Thus, some researchers reported that children’s attitudes toward out-groups were related to their parents’ explicit, rather than implicit, expression of out-group attitudes (Holub et al., 2011; Costello and Hodson, 2014). By contrast, others showed the effectiveness of implicit communication: parents’ implicit stereotyping facilitated children’s intergroup biases (Endendijk et al., 2013, 2014), whereas parents’ intergroup friendships reduced children’s intergroup biases (Vittrup and Holden, 2011; Pahlke et al., 2012). Explicit parent-child discussions of disabilities increase children’s knowledge regarding disabilities (Innes and Diamond, 1999) which, in turn, reduces the child’s intergroup biases (Magiati et al., 2002; Diamond and Huang, 2005; Diamond et al., 2008; Diamond and Hong, 2010; Gasser et al., 2014).

Children’s age may play a significant role in the relation between parents’ and children’s attitudes. For example, young children may have fewer opportunities to explicitly discuss intergroup biases with their parents because the latter do not believe their children are ready for such conversations (Pahlke et al., 2012; Hong et al., 2014). Moreover, young children may not be socially savvy enough to effectively process the implicit beliefs and attitudes communicated by their parents in daily interactions. Finally, older children may be more susceptible to social desirability concerns that would limit the explicit expression of prejudices and intergroup biases and make their explicitly expressed attitudes toward out-groups more similar to those of their parents who have been functioning under the same social desirability pressures. In accord with these notions, previous research found that children’s attitudes appeared to be more associated with parents’ attitudes as children become older, at least from the age of 5–6-years (Katz and Chamiel, 1989; Roberts and Lindsell, 1997; Hong et al., 2014).

### Parenting Styles

Parenting practices to a large extent affect children’s personality traits and attitudes toward others. Parenting can be classified according to responsiveness and control dimensions, resulting in four parental styles: authoritarian, authoritative, permissive, and uninvolved (Baumrind, 1994). ***Authoritarian*** parents are demanding, but not responsive; they promote over-control, obedience to authority, rigidity, and use of punishment. ***Authoritative*** parents show high responsiveness and high control; they are warm but demanding, they set rules and provide guidance, but also promote respect and autonomy. ***Permissive*** parents are warm and responsive, but not demanding; they do not set rules, but provide ample autonomy. ***Uninvolved*** parents are cold and undemanding; their children receive no warmth, no rules, and very little attention or guidance.

Research on permissive and uninvolved parenting styles in relation to children’s views and attitudes produced inconclusive results. By contrast, the authoritarian parenting style has been shown to be associated with conservative views in grown-up children, whereas authoritative parenting is associated with liberal views (Adorno et al., 1950; Jost et al., 2003; Oesterreich, 2005; Fraley et al., 2012; Wegemer and Vandell, 2020). In general, under-controlled children tend to grow up to be adults with liberal views, while over-controlled children often become conservatives (Block and Block, 2006). Strict, unaffectionate, and punitive parenting produces social conformists who perceive the world as hostile and threatening, promote authoritarian sociopolitical attitudes and are more likely to display intergroup biases (Duckitt et al., 2002; Holub et al., 2011; Costello and Hodson, 2014; Jugert et al., 2016).

Parents’ sociopolitical attitudes may be passed to their children via parental practices that shape specific personality traits. For example, parents with conservative views tend to enforce strict rules, discipline, and respect for authority (Lakoff, 1996; Wilcox, 1998; Barker and Tinnick, 2006; McAdams et al., 2008). These parental practices, in turn, are more likely to produce a fearful individual with low self-esteem, who may be protective of the in-group and discriminative toward out-groups. By contrast, parents with liberal views tend to be loving and empathetic; they foster the same loving, emphatic, accepting, and open-minded attitude in their children (Lakoff, 1996; McAdams et al., 2008); these grown-up children are more likely to condemn intergroup biases and social exclusion.

Furthermore, authoritative parenting, use of inductive reasoning, and healthy limit setting are all associated with higher levels of children’s empathy (Bryant, 1987; Janssens and Gerris, 1992; Krevans and Gibbs, 1996; Hoffman, 2000), whereas authoritarian parenting, excessive parental control, power assertion, and harsh punishment are associated with lower levels of children’s empathy (Hastings et al., 2002). Since empathy promotes the development of social skills (Diamond, 2001; Eisenberg et al., 2006; Mestre et al., 2019; Portt et al., 2020), authoritative parenting yields the best outcomes in terms of children’s emotional intelligence, social-behavioral skills, and social competence (Ladd and Pettit, 2002; Wang et al., 2019); the latter, in turn, may increase children’s positive peer relationships and acceptance of peers with disabilities (e.g., Diamond, 2001). By contrast, authoritarian parenting, which is related to decreased emotional understanding (Wang et al., 2019), may result in more negative attitudes toward peers with disabilities.

### Attachment Styles

Parental practices shape the individual’s attachment style, which further frames the individual’s future social attitudes and relationships. Parents serve as a secure base for infants to explore their environment while being protected from possible threats (Bowlby, 1969; Ainsworth, 1991). The level of availability, responsiveness, and supportiveness of a caregiver (the attachment figure) determines the social mental models that individuals use to build their relationships with important others during the lifetime (Bowlby, 1969; Hazan and Shaver, 1987; Cassidy, 1994; Trinke and Bartholomew, 1997; Fraley and Shaver, 2000; Shaver and Mikulincer, 2002; Bretherton and Munholland, 2008).

The security of the parent-infant attachment is usually tested in the Strange Situation paradigm (Ainsworth and Witting, 1969; Ainsworth and Bowlby, 1991). When being separated from the mother in the presence of a stranger in an unfamiliar setting, children show different responses in terms of seeking and maintaining contact with a caregiver, avoiding contact, or resisting contact (Strange Situation Classification; Ainsworth and Witting, 1969; Ainsworth and Bowlby, 1991). Infants’ dispositional differences in the Strange Situation are manifested in the following dichotomies: sociability vs. fear, affiliation vs. exploration, and approach vs. avoidance (Hatemi et al., 2013). Behaviors exhibited by infants in the Strange Situation classify them as having a secure, insecure anxious-ambivalent, or insecure avoidant attachment styles (Ainsworth et al., 1978).

***Securely attached*** infants actively explore their surroundings, maintain contact with the mother, approach the stranger, become distressed when separated from the mother and easily comforted upon her return (Ainsworth et al., 1978). As adults, these individuals are approach-oriented, dependable, and trustworthy (Hazan and Shaver, 1987; Collins, 1996). By contrast, ***avoidantly attached*** infants show less exploration and avoid contacting the mother or the stranger; they do not demonstrate strong positive or negative emotions upon the mother’s departure or return. Adults with avoidant/dismissive attachment style have a hard time trusting others and getting into close, intimate relationships. Finally, ***anxious-ambivalent*** infants show low levels of exploration, are reluctant to initiate contact with the stranger, become visibly distressed when separated from the mother, and display inconsistent emotions upon her return. As adults, they worry excessively about their relationships with others and tend to get too close to others, often scaring them away (Hazan and Shaver, 1987). Moreover, individuals scoring high on the anxiety dimension tend to have lower self-esteem and less positive self-views than their more securely attached peers (Bartholomew and Horowitz, 1991; Mikulincer, 1998).

The attachment style formed during infancy determines to a large extent the person’s future sense of security, social life, and worldviews (Kagan et al., 1988; Jost et al., 2003). Responsive parenting establishes a secure base for the exploration of environment and tolerance of novelty and uncertainty (Mikulincer and Shaver, 2001). In general, secure attachment is typically associated with liberalism, whereas insecure anxious-ambivalent attachment is linked to conservative views in grown-up children (Mikulincer, 1997; Mikulincer and Florian, 2000; Mikulincer and Shaver, 2001; Koleva and Rip, 2009; Wegemer and Vandell, 2020).

Secure attachment may enable people to embrace differences in the members of out-groups; even priming secure attachment (secure base schema) reduced negative evaluations of out-group members, irrespective of the individual’s underlying attachment style (Mikulincer and Shaver, 2001; Weise et al., 2008; Koleva and Rip, 2009; Gillath and Hart, 2010). By contrast, individuals with insecure attachment in interpersonal relationships tend to seek security in their affiliation with groups or institutions (Smith et al., 1999; Popper and Mayseless, 2007), which triggers intergroup biases and social exclusion of out-group members.

Furthermore, the feeling of vulnerability stemming from an insecure attachment may result in defensive stereotyping and exclusion of out-group members perceived as “strangers” during adulthood (Mikulincer, 1997; Mikulincer and Shaver, 2001; Hatemi et al., 2013). For example, people with higher levels of social anxiety would compare themselves to unfamiliar others using a greater number of attributes/dimensions and a greater number of comparisons per dimension (Antony et al., 2005), thus, being less likely to perceive similarity and in-group affiliation, and more likely to protect in-group through defense and support punitive policies against out-groups (Carney et al., 2008; Hatemi et al., 2013). In summary, parental responsiveness to an infant may determine the quality of the secure base that the individual would use in social relationships with others and shape the individual’s attitudes toward out-group members.

## Societal Factors Affecting Perception of Disability

In addition to personality and parental factors, societal factors add another layer of influences shaping children’s attitudes toward individuals with disabilities.

### Exposure

According to the “contact hypothesis” (Allport, 1954), prejudice may result from the incomplete or incorrect information about out-groups, which leads to overgeneralization and social exclusion; however, positive contact with out-group members may reduce stereotypes and intergroup biases. Familiarity with an out-group allows identification of similarities between the in- and out-group members, advances understanding of others, reduces anxiety and perceived out-group threat, as well as improves perspective taking and empathy (Dovidio et al., 2005; González and Brown, 2006; Pettigrew and Tropp, 2008; Pettigrew et al., 2011). In this case, inclusive education, that places typically developing children and those with disabilities in the same classroom, should reduce intergroup biases and improve attitudes toward children with disabilities.

Previous research comparing inclusive and non-inclusive classrooms, indeed, found that inclusion and exposure has positive effects on typically developing children’s attitudes toward and acceptance of peers with disabilities (Diamond and Carpenter, 2000; Nikolaraizi et al., 2005; Nowicki, 2006; Rillotta and Nettelbeck, 2007; Siperstein et al., 2007; Feddes et al., 2009; Kalyva and Agaliotis, 2009; Gasser et al., 2014). Face-to-face interactions increase children’s knowledge about disabilities and understanding of special needs and capabilities, as well as improve their attitudes toward peers with disabilities (Nikolaraizi et al., 2005; Gasser et al., 2014; Yildirim Hacıibrahimoğlu and Ustaoğlu, 2020). Importantly, even non-physical, imaginary exposure to out-group members has positive effects. Thus, reading stories, imagining or acting out contact and friendship with out-group members reduces children’s intergroup biases (Langer et al., 1985; Nesdale et al., 2005b; Cameron et al., 2006, 2011; Stathi et al., 2014; Vezzali et al., 2015). The duration of intervention may also be an important factor. For example, a several-week-long intervention involving reading stories about children with disabilities and participating in guided discussions improved young children’s attitudes toward individuals with disabilities (Cameron et al., 2007); whereas 1-hour-long intervention failed to reduce intergroup biases in young children (Gonzales et al., 2017).

Age is another factor influencing children’s response to inclusive settings and malleability of their attitudes toward peers with disabilities. After having face-to-face exposure to peers with disabilities, 7–10-year-old typically developing children reported more favorable attitudes than 11–16-year olds (Armstrong et al., 2016); similarly, 9-year olds showed higher level of positive attitudes and social inclusion than 12-year olds (Gasser et al., 2013). Thus, the most positive effect of exposure in the inclusive school context has been shown for elementary school students, rather than middle school ones (Krahé and Altwasser, 2006; Rillotta and Nettelbeck, 2007; Gasser et al., 2013), possibly because younger children’s attitudes toward out-groups are less stigmatizing and more malleable (Innes and Diamond, 1999; Bell and Morgan, 2000). Furthermore, early (elementary and middle school years) experiences in inclusive school environments may advance the development of children’s moral reasoning, making them more socially inclusive during high-school years (McDougall et al., 2004; Shalev et al., 2016).

Importantly, previous research reported not only positive, but also non-significant or even negative effects of exposure (Verkuyten and Kinket, 2000; Nowicki and Sandieson, 2002; Smith-D’Arezzo and Moore-Thomas, 2010; Kurtz-Costes et al., 2011; Vittrup and Holden, 2011; Pahlke et al., 2012; Vezzali et al., 2012; Huckstadt and Shutts, 2014; Aboud et al., 2015; Gibson et al., 2017; Ersan et al., 2020). Placement of children with disabilities in a general classroom does not automatically produce peer acceptance and social inclusion (Kluwin and Gonsher, 1994; McEvoy and Odom, 1996). Exposure to out-group members may create discomfort, insecurity, anxiety, and fear (Ward et al., 1994; Killen et al., 2013). For example, in typically developing 10–11-year-old children, those with intellectual disability induced feelings of sadness, pity, and sympathy (Beaulieu-Bergeron and Morin, 2016), as well as fear and anger (Nowicki et al., 2014). Negative feelings were associated with typically developing children’s focus on differences between them and children with disabilities, such as differences in social, emotional, and cognitive skills (Nowicki et al., 2014).

Note that interactions with out-group members may also be discouraged by the in-group; typically developing children are often concerned about their own social status among peers if they want to interact with children having disabilities (Kalymon et al., 2010; Obrusnikova et al., 2010). Therefore, typically developing children often accept their peers with disabilities only at a superficial level, with seemingly positive attitudes not being translated into readiness to interact and approach-oriented behaviors (Nikolaraizi and De Reybekiel, 2001). As a result, even in inclusive environments, children with disabilities may feel excluded and socially isolated because other children prefer to play with typically developing peers (Estell et al., 2009; Koster et al., 2010; Carvalho et al., 2014).

Negative attitudes often are based on misconceptions children have about peers with disabilities. For example, 5–7-year-old children expressed concerns that peers with disabilities may need medical care, be contagious, or just not be able to play (Nikolaraizi et al., 2005). Furthermore, learning disability was perceived by 10–11-year-old children not only as a limited mental capacity, but also as a character deficit: sign of laziness and lack of motivation to work harder (Smith-D’Arezzo and Moore-Thomas, 2010). Providing knowledge about different disabilities, both physical and intellectual, has become the focus of many interventions aiming at changing attitudes toward peers with disabilities in typically developing elementary school students (Favazza and Odom, 1997; Swaim and Morgan, 2001; Krahé and Altwasser, 2006; Holtz, 2007; Rillotta and Nettelbeck, 2007; Ison et al., 2010).

Furthermore, typically developing children may socially exclude peers with disabilities due to the nature of activities in which they participate. More social exclusion of children with disabilities is observed indoors rather than outdoors (Hong et al., 2020); outdoors likely provides more space to allow multiple playmates and encourage social interactions (Verhaegh et al., 2006). Children were also more exclusive of peers with disabilities during academic activities rather than play; play activities may provide more opportunities for children to engage in collaborative games (Hong et al., 2020). However, play activities requiring mobility resulted in more social exclusion of children with disabilities (Diamond and Tu, 2009; Diamond and Hong, 2010). Moreover, children were more likely to exclude peers with disabilities from academic or sport rather than social activities, likely because the group efficacy and threat of failure are more salient in the former type of activities (Gasser et al., 2014).

The effectiveness of exposure to individuals with disabilities on changing attitudes of typically developing children depends on the quality of the interactions (Skinner and Meltzoff, 2019). Positive changes in children’s attitudes were recorded when their contact with children having disabilities was regular, scaffolded by adults, and structured to advance understanding, reduce anxiety, as well as promote empathy, acceptance, interdependence, and cooperation rather than competition (Pettigrew and Tropp, 2000; Diamond, 2001; London et al., 2002; Kurtz-Costes et al., 2011; Kang and Inzlicht, 2012; Vezzali et al., 2012, 2015; Yu et al., 2012; Berger et al., 2015; Armstrong et al., 2016). Also, more frequent contact with peers having disabilities was associated with more positive attitudes (Favazza and Odom, 1997; Okagaki et al., 1998; Cameron et al., 2007; Hong et al., 2014). Importantly, the relation between contact and attitude is bidirectional: more structured, positive exposure to individuals with disabilities results in better understanding of disability and social acceptance, which, in turn, promotes further interest and willingness to interact (Hong et al., 2014).

### Cultural Differences

Culture represents a dynamic set of aspirations, values, beliefs, attitudes, and behaviors shared by a group of people and passed from one generation to another (Coleridge, 2000; Dickson et al., 2000; Matsumoto, 2001). Development of social attitudes and intergroup biases takes place within a cultural context; this is especially true in the case of disability since the latter is a socially constructed concept (Shweder and Sullivan, 1993; Coleridge, 2000; Gollnick and Chinn, 2002; Narayan, 2002; Mandell and Novak, 2005).

The definition of disability depends on the traits and capacities valued in a particular culture or social context (Whyte and Ingstad, 1995). For example, Tuareg in Sahara consider excessive freckles and small buttocks as impairment, since these features are socially disapproved and may prohibit marrying and, thus, fully participating in social life (Halatine and Berge, 1990). By contrast, on the island of Martha’s Vineyard in Massachusetts, deafness was not considered impairment, but rather as a normal human variation: over generations, individuals with hereditary congenital deafness were so common that the majority of hearing population became fluent in sign language, which allowed deaf residents to become fully integrated into society (Groce, 1985). These two examples demonstrate the way culturally shaped values arbitrarily define disability. Moreover, in “simple” societies, such as Martha’s Vineyard, where individuals have wide-spread kinship ties, regular face-to-face contact, considerable interconnection, and integration into community life, a single characteristic, such as a physical impairment, does not define one’s social identity (Scheer and Groce, 1988).

In contrast to simple societies, in complex societies individuals are not so interrelated; social relationships beyond the immediate family are often task-oriented and rather impersonal (Wright, 1983). In a complex society with a large number of impersonal social interactions, heuristics become handy to avoid cognitive overload; as a result, salient personal characteristics that deviate from the norm may define one’s social identity for an easy in-group vs. out-group classification and approach vs. avoidance behavior. Indeed, the 18–19th centuries’ industrialization and urbanization led to a more complex society and significantly increased social exclusion of individuals with disabilities (Stone, 1984).

Another feature of a society that may affect the level of social exclusion is individualism vs. collectivism. Individualistic societies promote respect for individual differences, values, and goals. By contrast, collectivistic societies value group goals and uniformity in the ways people look and think; such pressure for uniformity makes any deviation from the norm salient and negatively valenced. In individualistic societies, being “normal” has a neutral to slightly negative connotation: there is nothing special about a “normal” individual, which is boring. By contrast, in collectivistic societies, such as Japan, being normal is required for social approval and inclusion (Kuroishi and Sano, 2007; Yamada, 2009). Previous research found that collectivistic societies typically manifest less positive attitudes toward individuals with disabilities compared to individualistic societies (Black et al., 2003; Rao et al., 2010; Benomir et al., 2016; Huppert et al., 2019; Ersan et al., 2020).

Beliefs about the perceived cause of a disability to a large extent determine attitudes and behaviors toward individuals with disabilities within a family and society (Groce and Zola, 1993). For example, some societies (e.g., Navajo in US; Chagga in Tanzania; Ga in Ghana; some communities in Benin) perceive individuals with disabilities as divine beings, possessing sixth sense, protected by supernatural powers, or being pacifiers of the evil spirits; these beliefs result in awe, special care, kind treatment, and social inclusion of such individuals (Wright, 1960; Medina et al., 1998). On a negative note, in such communities, individuals with disabilities do not receive treatment for their impairment since it may question the God’s will or interfere with supernatural powers.

By contrast, other societies (e.g., Hopi in the United States; Ashanti in Ghana; Ainu in Japan; some communities in Mexico, Puerto Rico, Haiti, Nigeria, Kenya, Zimbabwe; Pakistan, India, China, Taiwan, Hong Kong) believe that disability is a result of parental sexual misconduct, sins conducted in previous life, witchcraft, juju, family curse, God’ punishment, or the involvement of evil spirits (Munro, 1963; Abosi and Ozoji, 1985; Groce and Zola, 1993; Cheng and Tang, 1995; Rogers-Adkinson et al., 2003). Shame associated with disability led to severe mistreatment and social exclusion of such individuals. The view of disability as a result of past transgressions prohibits access to resources, medical care, and special interventions for individuals with disabilities (Groce and Zola, 1993; Tsang et al., 2003).

Furthermore, the concepts of fairness, equality, and human rights differ significantly between traditional^6^ and modern societies: perception of disability as a divine punishment, fate, or karma seemingly justifies social exclusion and eliminates the necessity of intervention (Coleridge, 2000). Previous research showed that participants from China, Taiwan, and Hong Kong (traditional societies) had more negative attitudes and higher propensity of social exclusion toward individuals with disabilities than participants from the United States, United Kingdom, and Germany (modern societies) (Westbrook et al., 1993; Chan et al., 2002; Chen et al., 2002; Wang et al., 2003; Brown et al., 2009). Thus, in Asian countries, the impairment seems to become the single salient characteristic defining the identity and social life of the individual. Shame and stigma associated with disability alienates individuals with disabilities from the rest of the society, limits interpersonal contacts and opportunities to get more knowledge and understanding of disability for typically developing individuals.

However, being individualistic, developed, and modern, does not necessarily place a society among socially inclusive toward disability. For example, kindergartners from the Netherlands reportedly had much more negative attitudes toward peers with disabilities compared to children from the United States or Greece (de Boer et al., 2012a). A possible explanation to this phenomenon could be a lagging behind implementation of inclusive education programs in Dutch schools. Fewer opportunities to communicate with peers having disabilities may exacerbate intergroup biases and prevent social inclusion; instead, being perceived as different and unfamiliar, individuals with disabilities may be treated with caution, fear, and avoidance (Scheer and Groce, 1988).

Importantly, children acquire the ***culturally defined*** concepts of ability vs. disability through everyday interactions with peers and adults, as well as from the media (Hodkinson, 2007; Chen et al., 2012; Varenne, 2018). Depending on cultural beliefs, norms, and traditions, children are socialized in a particular way, which shapes their own beliefs, attitudes, and behaviors towards individuals with disabilities. For example, children socialized to respect individual differences may be more accepting of individuals with disabilities (Crystal et al., 1999; Shweder et al., 2007). By contrast, Japanese children are socialized to become highly sensitive to any differences that would potentially stigmatize themselves or others (Haight et al., 2016; Kayama et al., 2016). This sensitivity may have dual outcomes: on the one hand, it increases empathy and compassion, making people more understanding and willing to help; on the other hand, it leads to stigmatization, marginalization, and social exclusion (Sano and Kuroishi, 2005; Kayama and Haight, 2014; Sato et al., 2015; Kayama, 2017). Thus, many Japanese families with individuals having disabilities traditionally felt stigmatized and socially excluded by others (Sato et al., 2015), which resulted in hiding family members with disabilities and declining special education opportunities and services available to them (Tachibana and Watanabe, 2004; Jegatheesan, 2009; Kayama and Haight, 2014).

In summary, cultural differences in attitudes toward individuals with disabilities represent a multidimensional construct, which includes traditional values and socialization practices, causal beliefs about disability, collectivistic vs. individualistic tendencies, as well as religious traditions. Importantly, cultures are non-homogenous: different strata in the same society may have different beliefs, depending on the level of education and religious affiliations, among other factors. Moreover, cultures are dynamic: what was a cultural norm 20 years ago may not be such today; for example, the widespread implementation of inclusive education programs may dramatically change attitudes toward disabilities over one generation.

## Conclusion

The main purpose of the current review was to identify factors that affect perception of disability in the developmental context. When and how do children develop positive vs. negative attitudes toward individuals with disabilities? What factors are imperative in this developmental process?

The current review explored disability perception in the light of the ***in-group vs. out-group dichotomy***. Development of social identity shapes the individual’s beliefs about self and others, leading to classification of others into “us” vs. “them,” in-groups vs. out-groups (Allport, 1954; Tajfel and Turner, 1979; Hatemi et al., 2013). Attitudes toward out-groups are often infused with feelings of uncertainty, discomfort, anxiety, and fear (Ward et al., 1994; Killen et al., 2013). Lack of knowledge about an out-group may lead individuals to perceive it as a potential threat, triggering a self-protective defense reaction manifested in negative attitudes toward members of an out-group, stigmatization, and discrimination (Stephan and Stephan, 1993; Ybarra and Stephan, 1994). Thus, intergroup biases lead to social exclusion of out-groups and social isolation of their members. Since individuals with disabilities may be perceived as a special case of out-groups, the mechanisms involved in out-group perception should also apply to the perception of disability.

Previous research suggests that during the child’s ***development***, attitudes toward out-groups and individuals with disability become increasingly negative across 3–7-year period, but gradually improve thereafter (Averhart and Bigler, 1997; Bigler and Liben, 2007; Nesdale and Brown, 2004; Dunham et al., 2011; Raabe and Beelmann, 2011; Dunham and Emory, 2014; Baron and Dunham, 2015). An increase in intergroup biases and negative attitudes toward out-groups is associated with an increased sociocentric awareness and social cognition about in-groups vs. out-groups, as well as more solidified social identity, which leads to in-group favoritism and out-group derogation (Brewer, 1999; Aboud, 2003; Nesdale, 2004, 2008; Baron and Banaji, 2006; Rutland et al., 2007; Dunham et al., 2011; Buttelmann and Böhm, 2014; Dunham and Emory, 2014; Baron and Dunham, 2015). Change to more positive attitudes toward out-groups in general, and disability in particular, at the age of 7–8 years may be attributed to the following factors: (1) children’s increased knowledge about disability (Diamond et al., 1997; Diamond and Hong, 2010; Gasser et al., 2014); (2) children’s cognitive development shifting to the concrete operational stage, which allows critical thinking, perspective taking, lesser focus on most salient features, and a decrease in overgeneralization (Piaget, 1970; Gasser et al., 2014); and (3) development of moral reasoning, increasing children’s awareness of human rights, equality, and social justice during social evaluations (Fisher et al., 1998; Turiel, 1998; McDougall et al., 2004; Smetana, 2006; Gasser et al., 2014; Beaulieu-Bergeron and Morin, 2016; Shalev et al., 2016).

To provide a comprehensive model of disability perception, the current review explored ***cognitive, affective*,** and ***behavioral*** components of children’s attitudes. Previous research suggests that cognitive aspects of disability perception determine affective components, which, in turn, translate into behavioral outcomes. Thus, children with a better understanding of disability tend to have more positive attitudes toward individuals with disability (Katz and Chamiel, 1989; Okagaki et al., 1998; Magiati et al., 2002; Diamond and Huang, 2005; Diamond et al., 2008; Diamond and Hong, 2010; Gasser et al., 2014); positive attitudes, in turn, make children more likely to exhibit approach-oriented behaviors, initiating interactions with peers having disabilities, and practicing social inclusion (Diamond, 1993; Okagaki et al., 1998; Roberts, 1999; Roberts and Smith, 1999; Favazza et al., 2000; Gaad, 2004). Importantly, exposure to individuals with disabilities informs typically developing children’s knowledge about disability (Nikolaraizi et al., 2005; Gasser et al., 2014; Yildirim Hacıibrahimoğlu and Ustaoğlu, 2020), thus, establishing a bidirectional connection among the cognitive, affective, and behavioral aspects of disability perception.

Furthermore, following the principles of the ecological systems theory (Bronfenbrenner, 1992; Bronfenbrenner et al., 1994), the current review explored a multilevel structure of potential factors influencing perception of disability at the level of ***society***, ***family*,** and ***school*** environment, as well as the ***individual***. Importantly, disability is a socially constructed concept: the extent to which impairment becomes a disability depends to a large extent on the cultural norms and traditions (Shweder and Sullivan, 1993; Hall and Hill, 1996; Coleridge, 2000; Gollnick and Chinn, 2002; Narayan, 2002; Mandell and Novak, 2005). In turn, cultural norms and traditions affect attitudes toward disability which are broadcast by the media, exhibited by teachers in schools, and modeled by parents to their children. Then, school environment and parental practices shape children’s individual characteristics (e.g., temperament, empathy, sympathy, ToM, self-esteem) that affect their perception of disability.

In terms of ***cultural differences***, simple societies are more likely to produce positive attitudes toward disability since the individual’s impairment is perceived as one of many, and not the defining, characteristic of the individual (Groce, 1985). By contrast, complex societies may create more conditions in which the impairment becomes a disability, thus preventing the individual’s full participation in the society; the use of heuristics in such societies often triggers response to the most salient characteristic of the individual, which may be impairment (Wright, 1983). Moreover, individualistic societies typically exhibit more positive attitudes toward disability than collectivistic ones, since the former respect individual differences, whereas the latter impose pressure for uniformity (Black et al., 2003; Kuroishi and Sano, 2007; Yamada, 2009; Rao et al., 2010; Benomir et al., 2016; Huppert et al., 2019; Ersan et al., 2020). It seems that in both simple vs. complex and individualistic vs. collectivistic dichotomies, societal factors that make impairment a salient feature of the individual lead to more negative attitudes toward individuals with disabilities.

Cultural norms and traditions also determine ***parental practices*** that may shape children’s attitudes toward disability (Wu et al., 2002; Porter et al., 2005). In some countries (e.g., China), the authoritarian parenting style is very common, whereas others (e.g., the United States) widely promote authoritative parenting style (Chen et al., 1997; Majumder, 2016). Authoritarian parenting may produce insecurely attached children with low self-esteem, low levels of empathy and sympathy, and fearful temperament (Bryant, 1987; Janssens and Gerris, 1992; Krevans and Gibbs, 1996; Hoffman, 2000) – the individual characteristics associated with conservative views, avoidance-oriented behaviors, high level of intergroup biases, and negative attitudes toward out-groups and individuals with disabilities (Jost et al., 2003; Fraley et al., 2012; Wegemer and Vandell, 2020). By contrast, authoritative parenting may produce securely attached children with high self-esteem, high levels of empathy and sympathy, and expressive, social personality (Hastings et al., 2002) – the individual characteristics associated with liberal views, approach-oriented behaviors, low level of intergroup biases, and positive attitudes toward out-groups and individuals with disabilities (Ladd and Pettit, 2002; Jost et al., 2003; Fraley et al., 2012; Wang et al., 2019; Wegemer and Vandell, 2020). As a result, cultural factors may affect the prevalence of particular parenting practices which, in turn, shape individual characteristics and attitudes toward out-groups and disability.

Furthermore, cultural norms determine the availability of inclusive eduction and school-based interventions, which play an important role in shaping children’s perception of disability. School-based interventions are effective if they are structured, increase knowledge about disability, promote cooperation rather than competition, focus on similarities rather than differences between children, and are implemented in early childhood (Pettigrew and Tropp, 2000; Diamond, 2001; London et al., 2002; Kurtz-Costes et al., 2011; Kang and Inzlicht, 2012; Vezzali et al., 2012, 2015; Yu et al., 2012; Berger et al., 2015; Armstrong et al., 2016).

**FIGURE 1:**
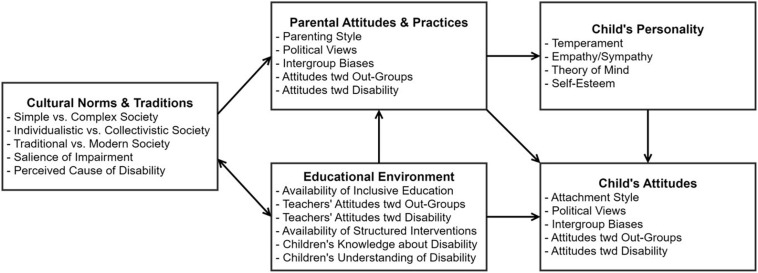
Integrative model providing a conceptual framework for understanding factors influencing the development of disability perception. The current model proposes that children’s attitudes toward disability may be influenced by a range of multi-dimensional factors encompassing different hierarchical levels of the child’s environment. Specifically, cultural norms and traditions guide parental practices and educational environment, which, in turn, shape children’s attitudes toward disability. In this process, parental practices interact with children’s personality traits. For example, an authoritative parenting style may promote secure attachment of the child, which would likely encourage higher self-esteem and empathy, both positively affecting the child’s attitude toward individuals with disability. At the same time, the child’s temperament (e.g., fearful and withdrawn vs. self-confident and social) might alleviate or exacerbate the effects of negative parental practices. Importantly, this model underlines the power of the educational environment to change not only children’s attitudes toward disability, but also parents’ intergroup biases and parental practices, as well as cultural norms in regard to disability perception.

A comprehensive review of the research allowed us to create an integrative model, encompassing complex relations among cultural, parental, and individual factors affecting perception of disability (Figure 1); this model may provide a conceptual framework for understanding the development of disability perception. We would like to emphasize here the power of education to change: (1) children’s knowledge, understanding, attitudes, and behaviors; (2) parental beliefs, attitudes, and practices; and even (3) cultural norms and traditions in respect to disability perception. Furthermore, for each level (cultural, parental, individual), we outlined specific factors that affect perception of disability in a positive vs. negative way (Table 1).

**TABLE 1:** The outline of factors that affect perception of disability in a positive vs. negative way.

**Factors**	**Effect on attitudes toward individuals with disabilities**	
	**Positive**	**Negative**
**Societal factors:**		
Simple/Complex	Simple	Complex
Individualistic/Collectivistic	Individualistic	Collectivistic
Traditional/Modern	Modern	Traditional
Valued …	Differences	Uniformity
Salience of impairment	Low	High
Cause of disability	Biological, divine	Transgressional
**Parental practices:**		
Parenting style	Authoritative	Authoritarian
Political views	Liberal	Conservative
Intergroup biases	Few	Many
**Educational environment:**		
Inclusive education	Available	Not available
Interventions	Available	Not available
Children’s knowledge about disability	High	Low
Children’s understanding of disability	High	Low
**Interventions:**		
Amount of structure	Structured	Unstructured
Promoting …	Cooperation	Competition
… Knowledge/Understanding	Increasing	Not increasing
Focus on …	Similarities between children	Differences between children
Setting	Outdoors	Indoors
Type of activities	Play	Academic
Mobility requirements	Low	High
Frequency of contact	High	Low
**Child’s personality:**		
Temperament	Social, expressive	Timid
Empathy/Sympathy	High	Low
ToM	Well-developed	Under-developed
Self-esteem	High	Low
**Child’s attitudes:**		
Attachment style	Secure	Insecure
Political views	Liberal	Conservative
Intergroup biases	Few	Many

In conclusion, future interventions, aiming to improve perception of disability during childhood and adolescence, should target not only educational, but also parental practices. We propose that parental education should be added as an important component of such interventions. Parents should understand that the way they treat their children early on will become the way their children will treat out-groups and individuals with disabilities later on: if parents are responsive to their children’s needs, show empathy and respect toward their children, promote autonomy, provide guidance, impose an adequate amount of control, and use inductive reasoning, their children will become self-confident and well-adjusted social beings, exhibiting high levels of empathy, social competence, and moral reasoning, which would translate into positive attitudes toward others, out-groups, and individuals with disabilities. Whereas people’s individual characteristics may determine their attitudes toward out-groups and disability, importantly, the former are shaped by parental practices and educational environment which, in turn, are the product of cultural norms and traditions.

## Strengths, Limitations, and Future Directions

The current review provides a comprehensive analysis of the contemporary research on the developmental aspects of disability perception that allows for deeper understanding of the ways in which cultural, parental, educational, and personality factors can either positively or negatively affect the formation of the individual’s emotional, cognitive, and behavioral aspects of disability perception. The proposed conceptual model of the disability perception development may guide future research on this topic. Based on this model, effective, age-appropriate interventions to improve perception of disability could be designed and tested.

Some factors potentially influencing the development of disability perception (e.g., genetic factors) were beyond the scope of this review. Moreover, some aspects of development (e.g., the embeddedness of emotions in language development; the role of early attachment to a caregiver, among the person’s other social relationships, in social development) discussed in the current review would require more deliberation due to controversies highlighted by previous research. Furthermore, previous research did not provide clear mechanisms behind some of the relations discussed in this review, such as the continuity of temperamental patterns across the person’s lifetime, the transition from early emotion mirroring to self-awareness of emotional states to empathy, and the potential role of puberty in the development of ToM. Extensive deliberation on these topics was beyond the scope of this review, but future research should address these issues.

## Author Contributions

IB conceptualized the manuscript. IB and EG wrote the manuscript. Both authors contributed to the article and approved the submitted version.

## Conflict of Interest

The authors declare that the research was conducted in the absence of any commercial or financial relationships that could be construed as a potential conflict of interest.

## Footnotes

1 The cognitive aspect reflects the individual’s thoughts, beliefs, and perceptions about an individual with disability; the affective aspect refers to the emotional valence of attitudes toward an individual with disability; the behavioral aspect highlights the individual’s willingness to interact with an individual having disability and the actual behavioral response.

2 Minimal social group paradigm is a methodology in which participants are randomly assigned to two arbitrary groups to study the minimal conditions for the emergence of the in-group favoritism and discrimination against out-group members.

3 Secure attachment is a parent–child emotional connection that satisfies the child’s need for security.

4 False belief test presents a child with the following scenario: two characters (e.g., Sally and Ann) are together in a room; Sally places an item in a specific location and leaves the room; meanwhile, Ann moves the item to a different location. Then, the child is asked where Sally will look for the item when she returns to the room. A child with ToM would point to the first location, while a child without ToM would point to the second location.

5 Authoritarian – imposing control over others, enforcing unquestioning obedience.

6 Traditional societies are characterized by powerful collective memories and importance of communal practices ensuring continuation of traditions, customs, and habits.
